# A cluster randomized stepped-wedge trial to de-implement unnecessary post-operative antibiotics in children: the optimizing perioperative antibiotic in children (OPerAtiC) trial

**DOI:** 10.1186/s13012-021-01096-1

**Published:** 2021-03-19

**Authors:** Sara Malone, Virginia R. McKay, Christina Krucylak, Byron J. Powell, Jingxia Liu, Cindy Terrill, Jacqueline M. Saito, Shawn J. Rangel, Jason G. Newland

**Affiliations:** 1 Brown School, Washington University in St. Louis, St. Louis, MO USA; 2 Division of Pediatric Infectious Diseases, Department of Pediatrics, Washington University in St. Louis School of Medicine, Campus Box 8116, 660 S. Euclid Ave, St. Louis, MO 63110 USA; 3 Division of Infectious Diseases, John T. Milliken Department of Medicine, Washington University in St. Louis School of Medicine, St. Louis, MO USA; 4 Division of Public Health Sciences, Department of Surgery, Washington University in St. Louis School of Medicine, St Louis, MO USA; 5 Division of Pediatric Surgery, Department of Surgery, Washington University in St. Louis School of Medicine, St. Louis, MO USA; 6 Department of Surgery, Boston Children’s Hospital, Harvard Medical School, Boston, MO USA

**Keywords:** De-implementation, Facilitation, Antibiotic stewardship, Cluster randomized stepped-wedge trial

## Abstract

**Background:**

Antibiotic-resistant infections have become a public health crisis that is driven by the inappropriate use of antibiotics. In the USA, antibiotic stewardship programs (ASP) have been established and are required by regulatory agencies to help combat the problem of antibiotic resistance. Post-operative antibiotic use in surgical cases deemed low-risk for infection is an area with significant overuse of antibiotics in children. Consensus among leading public health organizations has led to guidelines eliminating post-operative antibiotics in low-risk surgeries. However, the best strategies to de-implement these inappropriate antibiotics in this setting are unknown.

**Methods/design:**

A 3-year stepped wedge cluster randomized trial will be conducted at nine US Children’s Hospitals to assess the impact of two de-implementation strategies, order set change and facilitation training, on inappropriate post-operative antibiotic prescribing in low risk (i.e., clean and clean-contaminated) surgical cases. The facilitation training will amplify order set changes and will involve a 2-day workshop with antibiotic stewardship teams. This training will be led by an implementation scientist expert (VRM) and a pediatric infectious diseases physician with antibiotic stewardship expertise (JGN). The primary clinical outcome will be the percentage of surgical cases receiving unnecessary post-operative antibiotics. Secondary clinical outcomes will include the rate of surgical site infections and the rate of *Clostridioides difficile* infections, a common negative consequence of antibiotic use. Monthly semi-structured interviews at each hospital will assess the implementation process of the two strategies. The primary implementation outcome is penetration, which will be defined as the number of order sets changed or developed by each hospital during the study. Additional implementation outcomes will include the ASP team members’ assessment of the acceptability, appropriateness, and feasibility of each strategy while they are being implemented.

**Discussion:**

This study will provide important information on the impact of two potential strategies to de-implement unnecessary post-operative antibiotic use in children while assessing important clinical outcomes. As more unnecessary medical practices are identified, de-implementation strategies, including facilitation, need to be rigorously evaluated. Along with this study, other rigorously designed studies evaluating additional strategies are needed to further advance the burgeoning field of de-implementation.

**Trial registration:**

NCT04366440. Registered April 28, 2020, https://clinicaltrials.gov/ct2/show/NCT04366440.

**Supplementary Information:**

The online version contains supplementary material available at 10.1186/s13012-021-01096-1.

Contributions to the literature

- This study will evaluate the impact of two strategies, order set review and modification, and order set review and modification plus facilitation, as de-implementation strategies to eliminate unnecessary antibiotic use in children undergoing surgery.
- This study is one of the first to evaluate the impact of facilitation in de-implementation of unnecessary medical care, which will help inform specific tailored strategies for effective de-implementation in healthcare settings.
- This study, through its focus on reduction of inappropriate prescribing, will provide further insights into multi-level factors affecting de-implementation including the influence of the strength of the evidence-base, the impact of engaged clinical champions, and the organizational structures most effective.
- This study will demonstrate the importance of implementation science in advancing the field of antimicrobial stewardship.

## Background

Antibiotics have revolutionized healthcare by treating life-threatening infections and preventing infections for patients requiring needed surgical procedures. Unintended consequences of antibiotic use, worsened by unnecessary use, is the development of antibiotic resistant bacteria and *Clostridioides difficile* infections (CDI) which harm countless numbers of children every year [1–4]. The current rate of antibiotic resistant infections is now considered a major worldwide public health crisis by the World Health Organization [5, 6]. Significant antibiotic overuse occurs among children receiving unnecessary postoperative antibiotic prophylaxis. Although the Centers for Disease Control and Prevention’s (CDC) surgical site infection (SSI) prevention guideline do not recommend postoperative doses for low-risk procedures (clean or clean-contaminated) [7], empirical literature shows overuse occurs in up to 40% of surgical cases [8–10]. Furthermore, these excess doses put children at unnecessary risk. For example, even one additional dose of surgical prophylaxis in a child is associated with a sixfold increased risk for CDI [9]. Further, this overprescribing has not reduced the amount of post-operative complications in clean and clean contaminated surgical cases [11].

Consensus is building among professional organizations regarding the appropriate use of antibiotics to prevent SSIs while also mitigating overuse. In 2017, based on high-quality studies, CDC published SSI prevention guidelines strongly recommending to limit surgical antibiotic prophylaxis to one perioperative dose for clean and clean-contaminated cases [7]. The American Academy of Pediatrics (AAP) Committee on Infectious Diseases published five Choosing Wisely Items, the second of which stated not to “continue prophylaxis after the incision is closed for uncomplicated clean and clean-contaminated procedures” [12].

Antibiotic stewardship programs (ASP) interact with clinicians to help them prescribe antibiotics appropriately to limit the development of antibiotic resistant bacteria, reduce unnecessary adverse drug reactions, and prevent CDI. The CDC recommends that for hospital-based ASPs to be successful they should possess seven core elements [13] (Table 1). A key core element is that ASPs perform actions. These evidence-based actions/strategies include requiring clinicians to obtain approval prior to prescribing an antibiotic, prospectively auditing and providing feedback to clinicians after an antibiotic has been prescribed, and implementing guidelines to ensure the correct antibiotic is given at the correct dose for the correct duration. Frequently, ASPs target eliminating unnecessary durations of antibiotics such as postoperative antibiotics. An emerging body of literature highlights that unnecessary or inappropriate medical practices (e.g., post-operative prophylactic antibiotics) can be very difficult to eliminate [14]; however, evidence is converging to suggest that engagement of stakeholders, such as hospital administrators, departmental leadership, and clinicians, is essential to making meaningful progress toward reducing unnecessary medical practices [15–17].

**Table 1:** Core elements for hospital antibiotic stewardship programs

Core element	
Leadership commitment	
Accountability	
Pharmacy expertise	
Action	
Tracking	
Reporting	
Education	

The overall goal of the Optimizing Perioperative Antibiotic in Children (OPerAtic) trial is to identify the best strategy to de-implement unnecessary postoperative antibiotics in children undergoing low risk (i.e., clean and clean-contaminated) surgeries as recommended by the CDC and AAP. To achieve this goal, we will (1) develop two theoretically-informed strategies for reducing postoperative antibiotic prophylaxis in pediatric surgical cases considered low-risk for an SSI, and (2) compare the two strategies using a stepped-wedge cluster randomized research design in nine US children’s hospitals to determine efficacy and potential for implementation. In order to assess the above, we will (1) evaluate the impact of the de-implementation strategies on the rate of unnecessary postoperative antibiotic prophylaxis and on key clinical outcomes (e.g., SSI, CDI); and (2) assess each strategy by evaluating key implementation processes and outcomes (e.g., penetration). Ultimately, the enhanced uptake of evidence (antibiotic guidelines) via facilitation (de-implementation strategy delivered by ASP clinicians) will improve the clinical outcomes related to antibiotic use and CDI.

## Methods

### Study setting

This study will be conducted at nine tertiary-care children’s hospitals in the US that are members of the SHaring Antimicrobial Reports for Pediatric Stewardship (SHARPS) Collaborative and the National Surgical Quality Improvement Project-Pediatrics (NSQIP-P). The SHARPS Collaborative is comprised of over 70 hospitals that care for children dedicated to improving the use of antibiotics in all healthcare settings (http://pediatrics.wustl.edu/sharps). All hospitals from the SHARPS Collaborative in this study have an active ASP with dedicated financial support for a pediatric infectious diseases physician and pharmacist. Hospitals in NSQIP-P focus on improving the overall quality of surgical care through robust data collection performed by a dedicated data abstractor. Since 2008, hospitals in NSQIP-P have collected clinical and outcomes data, including rates of SSIs and CDI. Starting in July of 2018, prophylactic antibiotic use, including post-operative antibiotic use has been collected.

### Conceptual frameworks

**Fig. 1:**
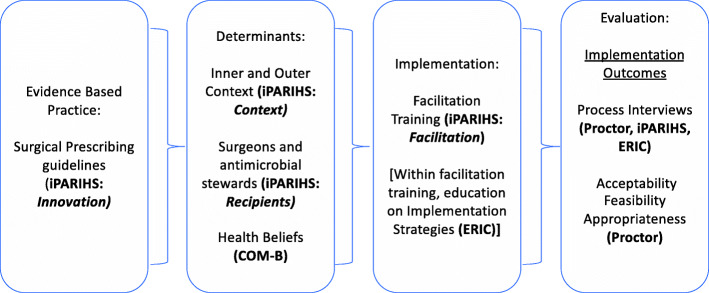
The use of iPARIHS, ERIC, COM-B, and Implementation Outcomes Framework in the development of the study design as well as their contributions to evaluation of implementation process and outcomes [18–21]

This study is guided by several conceptual frameworks (Fig. 1) that inform the identification and assessment of determinants [18, 19], implementation processes [19, 20], and the evaluation of implementation and clinical outcomes [21].

First, the Integrated Framework for Promoting Action on Research Implementation in Health Services (i-PARIHS) framework will inform our understanding of both implementation determinants and processes to de-implementation [19, 22]. i-PARIHS has been successfully used in a number of clinical settings [19, 23, 24], and its four domains are particularly well suited to inform determinants of de-implementation (i.e., the innovation, recipients, and context) and processes that may be necessary to ensure effective de-implementation (i.e., facilitation). *Innovation* describes the evidence and knowledge regarding a particular issue, as well as the qualities of the evidence that may influence how it is perceived by the potential user, recognizing that evidence may or may not be valuable to the end user based on local circumstances and priorities. *Recipients* are actors involved in (de-)implementation who may be affected by and influence the (de-)implementation of evidence. The COM-B framework [18] will be used to assess whether antimicrobial stewards have the capability, opportunity, and motivation to change order sets. *Context* characterizes the circumstances in which knowledge and innovation uptake occurs, consisting of multiple factors at the micro, meso, and macro levels. Finally, integral to this framework is *facilitation* as the active mechanism by which (de-) implementation of evidence occurs, making it an ideal fit for the current research proposal given that we are testing facilitation as a de-implementation strategy.

For this study, implementation of new evidence is facilitated by assessing and responding to both the evidence and recipients within their contextual setting. This requires an individual acting in a facilitating role (i.e., an antibiotic steward) with strategies and actions to enable evidence uptake and implementation. The Expert Recommendations for Implementing Change (ERIC) compilation [20] will be used to guide and categorize the implementation strategies used by antimicrobial stewardship teams. These strategies informed the facilitation training that will be provided to hospital teams in order to improve use of evidence-based implementation strategies, and will also be used to inform qualitative findings that will document the types of strategies that antimicrobial stewardship teams actually employ.

Finally, the Proctor and colleagues’ [21] Implementation Outcomes Framework will inform the selection and operationalization of implementation outcomes, such as acceptability, feasibility, appropriateness, and penetration. Additionally, iPARIHS, ERIC, and the Implementation Outcomes Framework will help inform coding of monthly qualitative interviews. These interviews will be coded using deductive content analysis in their respective domains: outcomes of practice change (Implementation Outcomes Framework), the process of facilitation and strategies that the teams utilize (iPARIHS and ERIC), and barriers to practice change (iPARIHS) [19–21, 25].

### Study design

**Fig. 2:**
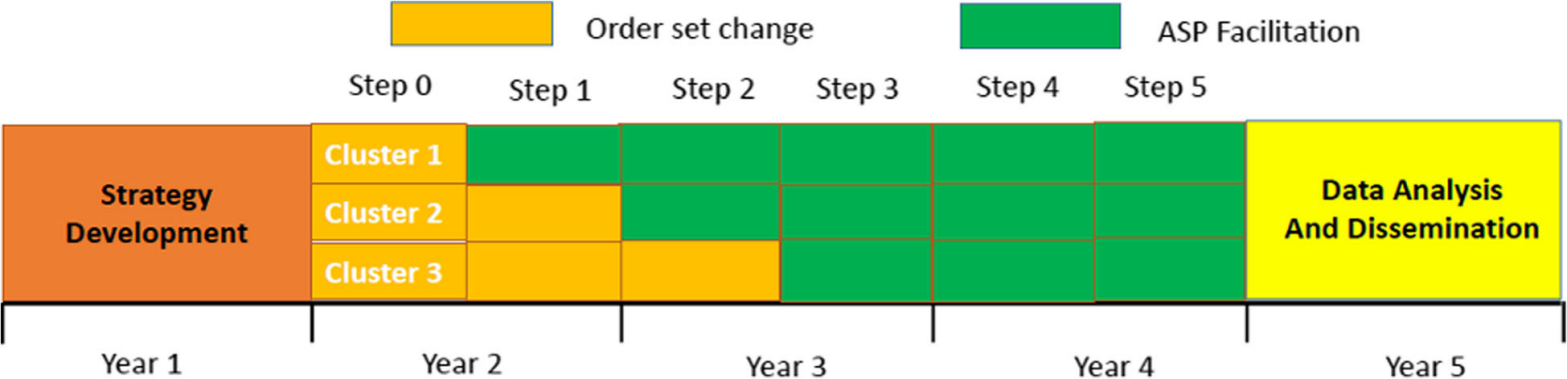
The proposed timeline for the cluster randomized stepped wedge designed study. Three clusters with 3 hospitals each will be included. All hospitals will start with the control strategy, order set change. Each step will be 6 months and starting in step 1, cluster 1 hospitals will receive the intervention strategy, facilitation training. By step 3, all hospitals will have received the intervention strategy

A stepped-wedged cluster-randomized trial will be conducted for 3 years (Fig. 2) [26]. We have selected this design because it (1) allows for effective implementation of the intervention at multiple sites across the study period, as synchronized implementation at a large number of sites can be difficult; (2) provides the best opportunity to thoroughly evaluate implementation and clinical outcomes since all sites will implement both interventions [27]; and (3) permits all participating hospitals to receive the ASP facilitation intervention, which will increase participation [28].

Each of the nine hospitals will be randomized into one of 3 clusters (cluster size = 3). At the beginning of step 0, all hospitals will implement the control intervention, order set review and modification. The experimental intervention—enhanced ASP facilitation of order set review and modification—will begin for cluster 1 hospitals at the start of step 1. The duration of each step will be 6 months. Hospitals in clusters 2 and 3 will begin the experimental intervention at the beginning of steps 2 and 3, respectively. The amount of time each cluster will receive the experimental condition will be 350 months (cluster 1), 24 months (cluster 2), and 18 months (cluster 3).

During each step, the ASP will attempt to eliminate unnecessary post-operative antibiotics through modifying order sets to remove unnecessary post-operative antibiotics in surgical procedures performed by surgeons primarily in the following specialties that submit data to NSQIP-P: general, neurosurgery, orthopedics, otolaryngology, plastics, and urology. During the experimental condition, the ASP teams will participate in a 2-day in-person facilitation workshop led by the study implementation scientist (VRM) and a pediatric infectious diseases physician with antibiotic stewardship expertise (JGN). This interactive workshop will provide the ASP teams’ knowledge on facilitation and the different strategies that can be used in their roles of facilitating order set change and modification.

The Washington University in St. Louis School of Medicine Institutional Review Board (IRB) has approved this study (IRB #:201912100) with a waiver of consent for children. An information sheet will be provided to the adult ASP team members and surgeons completing surveys. A single IRB is being utilized for this study.

### Study interventions

#### Control intervention (order set review and modification)

For this intervention, ASP teams participate in a brief 30 min webinar providing education about current evidence related to surgical prophylaxis and simple guidance on how to eliminate excess antibiotic doses. ASP teams are encouraged to obtain the electronic order sets used in post-operative cases among surgical specialties and modify them to eliminate any post-operative antibiotics in cases determined to be clean or clean-contaminated. If an order set does not exist for a surgical procedure, ASPs are encouraged to create an order set that does not have post-operative antibiotics. This intervention was chosen as a cost-effective strategy that has been effective in improving surgical antibiotic prophylaxis but has not been rigorously tested [17, 29]. Furthermore, existing literature indicates some kind of intervention over education alone is needed to prompt improvement [30]. Thus, we opted to select a relatively minimal intervention rather than no intervention at all.

#### Experimental intervention (enhanced ASP facilitation)

The experimental intervention will involve training the ASP teams in facilitation strategies in addition to conducting order set review and modification. We hypothesize that order set modifications will be more effective paired with facilitation training by fostering better engagement between ASP teams and surgeons, such as helping negotiate different communication styles and understanding of relevant data presentation. The facilitation guide will be based on the iPARIHS framework and education on implementation strategies informed by the ERIC framework [19, 20]. The study team will serve as external facilitators to train each ASP team (internal facilitators) to provide them the necessary skill sets to enhance their ability to modify the post-operative order sets. Each site will receive training in facilitation during a two-day workshop led by experts in implementation science (VRM) and pediatric antibiotic stewardship (JGN). The workshop leaders received facilitation training through the US Department of Veterans Affairs Quality Enhancement Research Initiative (QUERI) Behavioral Health Implementation Facilitation Training Hub [31].

The workshop led at each site will begin with assessing the team’s current success at changing order sets and review of the sites relevant clinical data from NSQIP-P. The ASP teams will then receive training in the following: communication, evidence presentation, capacity assessments, team building, use of implementation strategies, quality improvement methods, and disseminating information among multi-professional teams [32–34]. Role playing will be used to build and improve skills involving communication, team building, and conflict resolution. Quality improvement methods will be discussed focusing on the importance and utility of plan, do, study, act cycles [34–36]. The workshop will end on the topic of data presentation: the principles of evidence presentation and ways of presenting data. Following this, teams will have monthly phone calls to trouble shoot and discuss implementation and facilitation strategies.

#### Data collection

Using surveys, interviews, and clinical data, we will evaluate both implementation and clinical outcomes [21, 37]. In general, implementation outcomes will indicate the ease with which the proposed strategies can be implemented in a variety of contexts in the future. In combination with clinical outcomes (e.g., rates of unnecessary antibiotic use), we will test whether order set review and modification plus enhanced ASP facilitation provides additional benefit over the standard order set review and modification strategy.

### Baseline data

Prior to the start of the control intervention, hospital demographic information (e.g., bed size, surgical volumes, etc.) and the characteristics of the hospital’s ASP program (e.g., strategies utilized, locations where strategies implemented, and metrics utilized) will be collected. Additionally, this survey, will obtain specific information on efforts by the ASP at each site toward eliminating post-operative antibiotic use in the surgical subspecialties.

### Clinical data

Three-months following each step, clinical data for the 6 months of the step will be obtained from NSQIP-P. These data will include post-operative antibiotic use, surgical site infection, and *Clostridioides difficile* infection. This 3-month delay allows time for data collection and monitoring for complications including SSIs and CDIs. These measures are the same in both the control and intervention phase of the trial.

At each hospital, data collection will occur for 35 cases every 8 days, thereby providing clinical and outcome data for up to 1500 cases per year. Hospitals will preferentially sample up to 4 cases of the following high-volume cases: appendectomies, ventriculoperitoneal shunt placements, spinal fusions, ureteral re-implantation, and tracheostomy placement in children less than 2 years of age. Given current estimates of case volume and case mix, the “typical” participating hospital will collect about 8-12 of these up-sampled cases. The remaining cases will be randomly sampled across the 640 CPT codes until a total of 35 cases are included for those 8 days. While this sampling will not collect all cases, studies of appendectomy cases showed that NSQIP-P data were representative of the entire institutional experience [38].

Surgical procedures collected by NSQIP-P data abstractor at the hospitals participating are eligible for inclusion if they have a wound class of clean or clean-contaminated. Children undergoing surgeries due to infection will be excluded.

Data elements collected for each surgical case include demographics (e.g., age, sex, date of procedure), type of procedure, wound class (clean, clean-contaminated, contaminated, or dirty), co-morbidities, and resources utilized for the surgical case. Antibiotic prophylaxis data collected will include type of antibiotic, appropriate perioperative timing (i.e., within one hour prior to incision), whether redosing occurred, and duration of postoperative antibiotic prophylaxis.

The primary clinical outcome will be the percentage of clean and clean-contaminated cases that receive unnecessary post-operative antibiotics. Secondary clinical outcomes will include the rate of SSIs and CDIs.

### Implementation data

1. Process: Semi-structured interviews (see Supplemental file A for interview guide) will be conducted with a member of the ASP team at each site monthly through the duration of the trial. These interviews will be conducted virtually via zoom and will last ~30 min. We will assess the progress of the site in identifying, reviewing, and changing current surgical order sets and implementing new order sets that do not contain post-operative orders for antibiotics. Furthermore, information will be obtained regarding current barriers, and additional work being done to eliminate unnecessary post-operative antibiotics (e.g., stakeholder meetings).
2. Outcomes:
  - *Acceptability, Appropriateness, Feasibility*: We will assess the acceptability, appropriateness, and feasibility of each strategy using psychometrically strong and pragmatic survey measures [39], which will be distributed once during each step. All members of the ASP team will complete these surveys regarding the intervention(s) currently being implemented by the ASP. Therefore, during control, participants will complete the surveys for acceptability, appropriateness, and feasibility in relation to changing order sets as an implementation strategy. During the intervention, participants will complete separate surveys to independently evaluate changing order sets and the use of facilitation techniques.
  - *Penetration* will be measured as the number of order sets modified or newly created. These data will be collected monthly through the web-based semi-structured interview.
  - *Health beliefs*: Once per step, each member of the antibiotic stewardship team will be asked three health belief questions modeled off the COM-B system [18]. These questions assess the participants’ belief in their capability, motivation, and opportunity to change post-operative order sets.

## Statistical analysis plan

### Clinical outcomes

#### Primary hypothesis

Our primary hypothesis is that the experimental intervention of enhanced ASP facilitation will decrease the percentage of cases receiving unnecessary postoperative antibiotic prophylaxis from baseline more than the control intervention. To test this hypothesis, statistical model (1) will be used. With each cross-section consisting of *m* patients, we assume a model in which the logit of outcome for patient *i* at times *t* = 0, ⋯, *T* in hospital *k* = 1, ⋯, *K*, and cluster *l* = 1, 2, 3 is

1 $$ \mathrm{logit}\left({\pi}_{itkl}\right)=\gamma +{\theta}_t+{A}_{lt}\delta +{x}_{kl}+{h}_{tkl}+{\varepsilon}_{itkl}, $$

where *π*_*itkl*_ is probability of percentage of unnecessary postoperative antibiotic prophylaxis, ${\varepsilon}_{itkl}\sim N\left(0,{\sigma}_{\mathrm{error}}^2\right)$, ${h}_{tkl}\sim N\left(0,{\sigma}_{\mathrm{time}\mid \mathrm{clinic}}^2\right)$, ${x}_{kl}\sim N\left(0,{\sigma}_{\mathrm{clinic}}^2\right)$, with *ε*_*itkl*_, *h*_*tkl*_, and *x*_*kl*_ are all independent of one another, and

$$ {A}_{lt}=\left\{\begin{array}{c}1\ \mathrm{if}\ \mathrm{cluster}\ l\ i\mathrm{s}\ \mathrm{receiving}\ \mathrm{the}\ \mathrm{intervention}\ \mathrm{at}\ \mathrm{time}\ t\\ {}0\ \mathrm{if}\ \mathrm{cluster}\ l\ i\mathrm{s}\ \mathrm{receiving}\ \mathrm{the}\ \mathrm{control}\ \mathrm{at}\ \mathrm{time}\ t\kern2.5em \end{array}\right. $$

This hierarchical, multilevel model includes a fixed effect of time *θ*_*t*_, we set *θ*_0_ = 0. The parameter *δ* is the treatment effect; we assume that the treatment effect is maintained once the intervention has been initiated. The fixed effect *θ*_*t*_ must be estimated independently of the treatment effect so that a systematic change over time is not mistaken for an effect of treatment. The model also includes random effect, variation between clusters (*x*_*kl*_), and variation between times within a hospital (*h*_*tkl*_).

Unadjusted analysis through model (1) will be conducted, and an adjusted model will be developed. The following data elements will potentially be included in this model: hospital, age, surgical subspecialty (e.g., orthopedics, ENT, etc.), type of procedure, duration of hospitalization, and co-morbidities. The estimated intervention effect will be reported as odds ratio with 95% CIs and *p* values. The tests will be two-sided and the significance level will be set at 0.05. The statistical package SAS 9.4 will be used for all statistical calculations.

#### Secondary hypotheses

We will also test multiple secondary hypotheses to understand the additional impact of our strategy implementation. These include the following:

- The rate of surgical site infection will be no different prior to the study versus order set and/or enhanced facilitation groups.
- The rate of *Clostridioides difficile* infection will be improved when comparing control versus baseline, and experimental condition versus control or baseline.

A statistical model analogous to model (1) will be used for the secondary outcomes analyses, where *π*_*itkl*_ will be replaced by the rate of SSI or CDI and implementation outcomes, respectively.

### Implementation outcomes

A descriptive analysis will be conducted for the implementation outcomes. This study is not powered to assess group differences but rather to understand the pre-post facilitation training differences in implementation outcomes of feasibility, acceptability, and appropriateness. This evaluation will utilize a mixed methods design with a Qualitative + QUANITATIVE structure that serves the function of for complementarity [40].

The qualitative aspect of the study allows for an understanding of the processes associated with the study. The monthly phone calls will be coded using a deductive content analysis [25]. Identifying information from the interviews will be removed and the interviews will be coded based on context, facilitation and strategies, and outcomes. This will be informed by iPARIHS (context and facilitation), ERIC (strategies), and outcomes (Proctor) to develop the codes. The codes will allow for information to be indexed in these relevant areas of the study and will facilitate exploration of the relevant contextual factors, interventions, and outcomes related to the study. These interviews will be co-coded by two coders in order to increase reliability of coding. Any discrepancies in coding will be resolved through meetings and discussions. Further, any order sets developed or changed will be captured and counted through these monthly phone calls.

The generalized estimating equation (GEE) model with appropriate link function will be used to analyze the implementation outcomes, in which the correlation among the ASPs from the same cluster need to be considered. The autoregressive of first order as working correlation structure will be considered. The GEE model includes the group indicator, month, and the interaction term between group and month. The *p* values of the interaction term from type 3 analysis in the GEE model will be estimated to assess whether the outcome across all ASPs between two groups are different. Least square means for each outcome for each group will be estimated and the standard errors will be calculated within the use of GEE sandwich method when accounting for within-cluster correlation.

#### Power estimates

The sample size calculation is based on the primary outcome only. We utilized the sample size formula for repeated cross-section stepped-wedge cluster randomized trials from Hooper and colleagues [41]. This formula depends on the intra-cluster correlation (ICC), cluster auto-correlation, number of groups, number of steps, and the number of patients at each cross-section, where cluster auto-correlation is defined as the correlation between two population means from the same cluster at different times.

Our prior work from 32 children’s hospitals determined rates of inappropriate postoperative antibiotic use with an ICC of 0.121 and cluster auto-correlation of 0.328 [42]. The proposed experimental intervention is expected to decrease unnecessary postoperative antibiotic use rates from 33% to 13-18%. A total sample of 46 cases per step per hospital and 9 hospitals achieves 80% power to detect the inappropriate antibiotic rate difference of 0.20 between intervention (13%) and control groups (33%) at the significance level of 0.05, assuming an ICC of 0.121 and cluster auto-correlation of 0.328.

## Discussion

The proposed research will develop and evaluate de-implementation strategies to eliminate unnecessary postoperative antibiotic use in pediatric surgical cases. Two strategies, order set review and modification, and order set review and modification plus enhanced facilitation, will be compared. These strategies have been successful in previous healthcare implementation efforts but have not been directly compared [19, 43–46]. As each hospital receives their facilitation training, we hypothesize that participants’ beliefs in this process will improve. Important clinical outcomes including antibiotic use, CDI infection, and SSI infection will be assessed. If effective, these de-implementation strategies will support the reduction of overall antibiotic overuse, decrease the incidence of CDI, and will help maintain the effectiveness of our antibiotic arsenal for the future. The insights and information learned will help in the development of future strategies to de-implement other unnecessary medical practices.

De-implementation has become a prevalent area of study in implementation science, but there remains a lot to be learned about de-implementation mechanisms and strategies, especially in healthcare settings [47, 48]. This study will contribute to the implementation science literature in several ways. First, there is a need for more implementation and de-implementation strategies that are systematically designed and informed using relevant theories and frameworks [49–51]. This study will demonstrate how a number of frameworks, namely, i-PARIHS and COM-B, can inform the development and execution of a facilitation-based implementation strategy.

Second, while there is growing evidence for strategies that can influence the de-implementation off harmful and/or low-value care, there is a need for more experimental evaluations of de-implementation strategies, particularly multisite trials that rigorously evaluate their effects [52].

Third, while there is evidence that the facilitation can be an effective implementation strategy [45, 53], it has not, to our knowledge, been applied to de-implement low-value or harmful practices. This study will provide an example of how facilitation can be applied to promote de-implementation, but the evaluation of implementation processes will contribute to the facilitation literature by carefully tracking strategies used by facilitators and ASPs at each of nine implementation sites. Carefully tracking and reporting implementation strategies has been identified as a top priority for the field [51, 54].

Fourth, Norton and Chambers [48] note that like with implementation, de-implementation strategies will need to be designed to be responsive to multilevel barriers but also be feasible, adaptable, and generalizable to other settings. Facilitation as a de-implementation strategy is potentially well-suited to meet those criteria, because facilitators can efficiently ensure that the actual strategies deployed will be tailored to the strengths and needs of their contexts. This relatively adaptive approach is also consistent with the notion of equifinality, as there are multiple potential pathways to successfully de-implementing harmful or low-value care [55]. We will assess the acceptability, appropriateness, and feasibility of the de-implementation strategies used in this trial with psychometrically strong and pragmatic measures [39].

Finally, an open question in implementation science is whether implementation and de-implementation are distinct enough in their needs to require very different strategies. Recent evidence from Patey and colleagues [56] suggests that different behavior change techniques were used in implementation and de-implementation efforts. While this study is not directly comparing implementation and de-implementation efforts, our efforts to carefully track the implementation strategies used at each site will contribute to a better understanding of the types of strategies that are required for de-implementation and if/how they are different from typical implementation strategies identified in the literature.

A strength of this study is the use of a cluster randomized stepped-wedge design [26]. This design allows for a greater number of hospitals to receive both the control and intervention arms of the study than would otherwise be feasible with a standard randomized controlled trial. Furthermore, hospitals will be evaluated over 3 years, allowing for a better understanding of long-term effects of these interventions.

Several limitations are present in this study. First, the specific clinical outcomes from surgeries impacted by our implementation strategies may not be directly followed. Rather, we will be capitalizing on a separate data collection effort, NSQIP-P. While this means that some surgeries impacted by implementation strategies will be assessed, some will not, which may decrease the effect size observed. Second, this study will be unable to determine the direct impact of the de-implementation of postoperative antibiotic prophylaxis on antibiotic resistance, as SSI rates are infrequent, and the amount of time needed to discern a change in bacterial resistance patterns is much longer than the duration of this study. Lastly, this study will be done in a tertiary-care children’s hospitals and therefore may not be generalizable to other clinical settings such as community hospitals or outpatient clinics.

The addition of facilitation training of the ASP team may not result in a greater reduction of unnecessary postoperative antibiotic use than order set review and modification alone. If this occurs, an interrupted time-series analysis will be conducted evaluating the baseline data until the implementation of the order set to determine if the order set change impacted the use. Since order set change and modification is a simpler, less resource intense intervention, this result would be favorable and allows for more rapid dissemination. An important strength of the study is the use of the SHARPS Collaborative and NSQIP-P which will allow for immediate dissemination of the results to many additional hospitals.

Rigorously assessing de-implementation strategies is essential in providing high-value medical care. As clinical innovations advance medicine, routine, common practices that lack benefit and result in harm must be eliminated. The challenges to de-implementing evidence-based guidelines are different and therefore, need dedicated trials, like the OPerAtiC trial, to be conducted.

## Supplementary Information

**Additional file 1: Supplementary file A**. Semi Structured Monthly Interview Guide.

## Data Availability

Not applicable.

## Abbreviations

SHARPS: SHaring Antimicrobial Reports for Pediatric Stewardship
NSQIP-P: National Surgical Quality Improvement Project-Pediatrics
ASP: Antibiotic Stewardship Program
iPARIHS: Integrated Framework for Promoting Action on Research Implementation in Health Services
