# Supplementary material for: A cluster randomized stepped-wedge trial to de-implement unnecessary post-operative antibiotics in children: the optimizing perioperative antibiotic in children (OPerAtiC) trial
Source: Implement Sci. 2021 Mar 19;16:29. doi: 10.1186/s13012-021-01096-1 (PMC7980649; doi:10.1186/s13012-021-01096-1)
Supplement: Supplementary file 1 — Additional file 1: Supplementary file A. Semi Structured Monthly Interview Guide. [file 13012_2021_1096_MOESM1_ESM.docx]

Semi Structured Monthly Interview Guide

| **ORDER SET CHANGE QUESTIONS** | | |
| --- | --- | --- |
|  | Have you done anything to initiate or continue the process of changing order sets this month? |  |
| 2. | Have you identified order sets to change? | ❑ yes ❑ No If yes, how many? (numerical 1-5) |
| **If yes, ask the following questions for each order set identified.** | | |
| 2a. | Surgery/Procedure name: |  |
| 2b. | Were post op antibiotics on them? | ❑ yes ❑ No If yes, which one(s)? |
| 2c. | What duration | ❑ No antibiotics  ❑ 24 hours  ❑ 48 hours  ❑ >/= 72 hours |
| 2d. | Did you meet with the surgical team impacted by order sets? | ❑ yes ❑ No If yes, which one(s)? |
| 2e. | Have you changed the duration of any post op antibiotics from these order sets? | ❑ removed ❑ shortened  ❑ remained the same |
|  | If removed or shortened, which antibiotics?  If removed or shortened, what was the date of change completion?  If shortened, to what duration? |  |
| 2f. | Will you provide us a screen shot of the revised order set(s)?  For interviewer: If yes, please ask the team member to send the screen shot within the week to sara.malone@wustl.edu. | ❑ yes ❑ No Upload screen shot here: |
| 3. | Have you helped initiate any new surgical order sets that do not have post operative antibiotics included? | ❑ yes ❑ No If yes, which one(s)? |
| 3a. | Surgery/Procedure name? |  |
| 3b. | Will you provide us a screen shot of the revised order set(s)?  For interviewer: If yes, please ask the team member to send the screen shot within the week to sara.malone@wustl.edu. | ❑ yes ❑ No If yes, please upload |
| 4. | Other than order set changes, what else have you done this month to eliminate unnecessary post-operative antibiotic use? |  |
| 5. | Has anything happened (positive or negative) that would affect your ability to eliminate unnecessary post-operative antibiotics? |  |
| 6. | Is there anything else you feel like we need to know regarding post-operative antibiotic use at your hospital in the past month? | ❑ yes ❑ No If yes, please describe |

Site name: Person providing answers:

Completed by: Date completed:

| **FACILITATION PROCESS QUESTIONS (once in the intervention phase)** | | |
| --- | --- | --- |
| 1. | Have you leveraged or currently leveraging any of the facilitation strategies this month? | ❑ yes ❑ No If yes, which ones? |
| 2. | Have you performed or currently performing PDSA cycles? | ❑ yes ❑ No If yes, please describe |
| 3. | Have you used any of the team building techniques? | ❑ yes ❑ No If yes, please describe |
| 4. | Have you used any of the communication techniques? | ❑ yes ❑ No If yes, please describe |
| 5. | Have you performed a context assessment? | ❑ yes ❑ No If yes, please explain: |
| 7. | Have you done anything that is affecting your ability to eliminate unnecessary post-operative antibiotic use? | ❑ yes ❑ No If yes, please explain: |
| 8. | Has anything happened (positive or negative) that would affect your ability to eliminate unnecessary post-operative antibiotics? | ❑ yes ❑ No If yes, please explain: |

Site name: Person providing answers:

Completed by: Date completed:
